# Factors influencing first-time mothers’ introduction of complementary foods: a qualitative exploration

**DOI:** 10.1186/s12889-015-2250-z

**Published:** 2015-09-22

**Authors:** Anne Walsh, Lauren Kearney, Nicole Dennis

**Affiliations:** School of Nursing, Institute of Health and Biomedical Innovation, Queensland University of Technology Kelvin Grove, Brisbane, 4059 Queensland Australia; School of Nursing, Midwifery and Paramedicine, University of the Sunshine Coast, Sippy Downs campus, Sippy Downs, 4556 Queensland Australia; School of Exercises and Nutrition Science, Institute of Health and Biomedical Innovation, Royal Children’s Hospital Brisbane, Brisbane, Queensland Australia

**Keywords:** Parent, Complementary food, Solids, Decision making, Theory of planned behaviour, Information sources

## Abstract

**Background:**

Optimal infant nutrition comprises exclusive breastfeeding, with complementary foods introduced from six months of age. How parents make decisions regarding this is poorly studied. This study begins to address the dearth of research into the decision-making processes used by first-time mothers relating to the introduction of complementary foods.

**Methods:**

This qualitative explorative study was conducted using interviews (13) and focus groups (3). A semi-structured interview guide based on the Theory of Planned Behaviour (TPB). The TPB, a well-validated decision-making model, identifies the key determinants of a behaviour through behavioural beliefs, subjective norms, and perceived behavioural control over the behaviour. It is purported that these beliefs predict behavioural intention to perform the behaviour, and performing the behaviour.

A purposive, convenience, sample of 21 metropolitan parents recruited through advertising at local playgroups and childcare centres, and electronically through the University community email list self-selected to participate. Data were analysed thematically within the theoretical constructs: behavioural beliefs, subjective norms and perceived behavioural control. Data relating to sources of information about the introduction of complementary foods were also collected.

**Results:**

Overall, first-time mothers found that waiting until six months was challenging despite knowledge of the WHO recommendations and an initial desire to comply with this guideline. Beliefs that complementary foods would assist the infants’ weight gain, sleeping patterns and enjoyment at meal times were identified. Barriers preventing parents complying with the recommendations included subjective and group norms, peer influences, infant cues indicating early readiness and food labelling inconsistencies. The most valued information source was from peers who had recently introduced complementary foods.

**Conclusions:**

First-time mothers in this study did not demonstrate a good understanding of the rationale behind the WHO recommendations, nor did they understand fully the signs of readiness of infants to commence solid foods. Factors that assisted waiting until six months were a trusting relationship with a health professional whose practice and advice was consistent with the recommendations and/or when their infant was developmentally ready for complementary foods at six months and accepted them with ease and enthusiasm. Barriers preventing parents complying with the recommendations included subjective and group norms, peer influences, infant cues indicating early readiness and food labelling inconsistencies.

## Background

One of the many dilemmas experienced by first-time breastfeeding mothers is whether and when to introduce complementary foods to their infant’s diet. Global recommendations from the World Health Organisation (WHO) [1] and Australian National Health and Medical Research Council (NH&MRC) [2] currently advise exclusive breastfeeding until six months, with the introduction of nutritious solid foods to complement ongoing breastfeeding [1–3]. There is strong evidence supporting the importance of exclusive breastfeeding in reducing infant mortality and morbidity [4, 5], however, many mothers have concerns about the adequacy of their infant’s diet and for some, exclusive breastfeeding may be difficult due to other factors, such as return to paid employment or concerns about breastmilk supply. For the purpose of this study, complementary foods (CF) are defined as “all solid and liquid foods other than breastmilk or infant formula” [5]. Findings from this study will assist in our understanding of, and answer the research question ‘What decision-making processes are used by first-time mothers when determining the timeliness of introducing CF to their infant.’

There is broad agreement among health professionals that CFs should not be introduced before four months due to the displacement of breastmilk with foods with lower nutritional quality [5], and increased risk of allergy development [6]. Yet, by six months infants require their milk diet to be supplemented, specifically their iron and zinc stores [7–9]; two elements known to significantly influence infant and childhood growth and development [7]. Nutritionally, the first two years of life is critical, this is a time of rapid growth and development [8]. By six months of age the majority of infants are developmentally ready to manage CFs, both in terms of motor and digestive/gastrointestinal development [10]. To date, there is no demonstrated health benefit to the introduction of CFs prior to six months of age [1–3], and evidence suggests that early (prior to four months of age) introduction may place undue stress on the immature renal, digestive and immune systems [11]. Increased body fat and risk of respiratory and diarrhoeal illness and allergy have also been associated with early introduction of CFs [9].

Despite strong evidence supporting exclusive breastfeeding until six months, current Australian data suggests many parents introduce CFs earlier than six months. In Australia, in 2010 at least 35 % of infants aged 4 months, and 92 % of infants aged 6 months had consumed soft/semi-solid food [12], with exclusive breastfeeding rates in Queensland (where this study was conducted) even lower, only 9.5 % at five months of age; 90.5 % had consumed some form of CF [13]. Similarly a recent West Australian study found the median infant age for introduction of CF was four months, with 93 % of the cohort having received CFs by six months of age [14].

While these data provide a clear picture of current practice, they do not explore the complexity of social, psychological and cultural factors that may be influencing when, and to what extent, mothers choose to introduce complementary feeding. Some of these factors may be the same as those known to influence parental decision making in relation to other issues such as fever management [15, 16], health promoters [17], intention to breast feeding [18]; for example, knowledge, attitudes, social referents, and perceptions of control. In the Australian context, no theoretically based studies have been published qualitatively exploring these factors in relation to introduction to CFs.

**Fig. 1:**
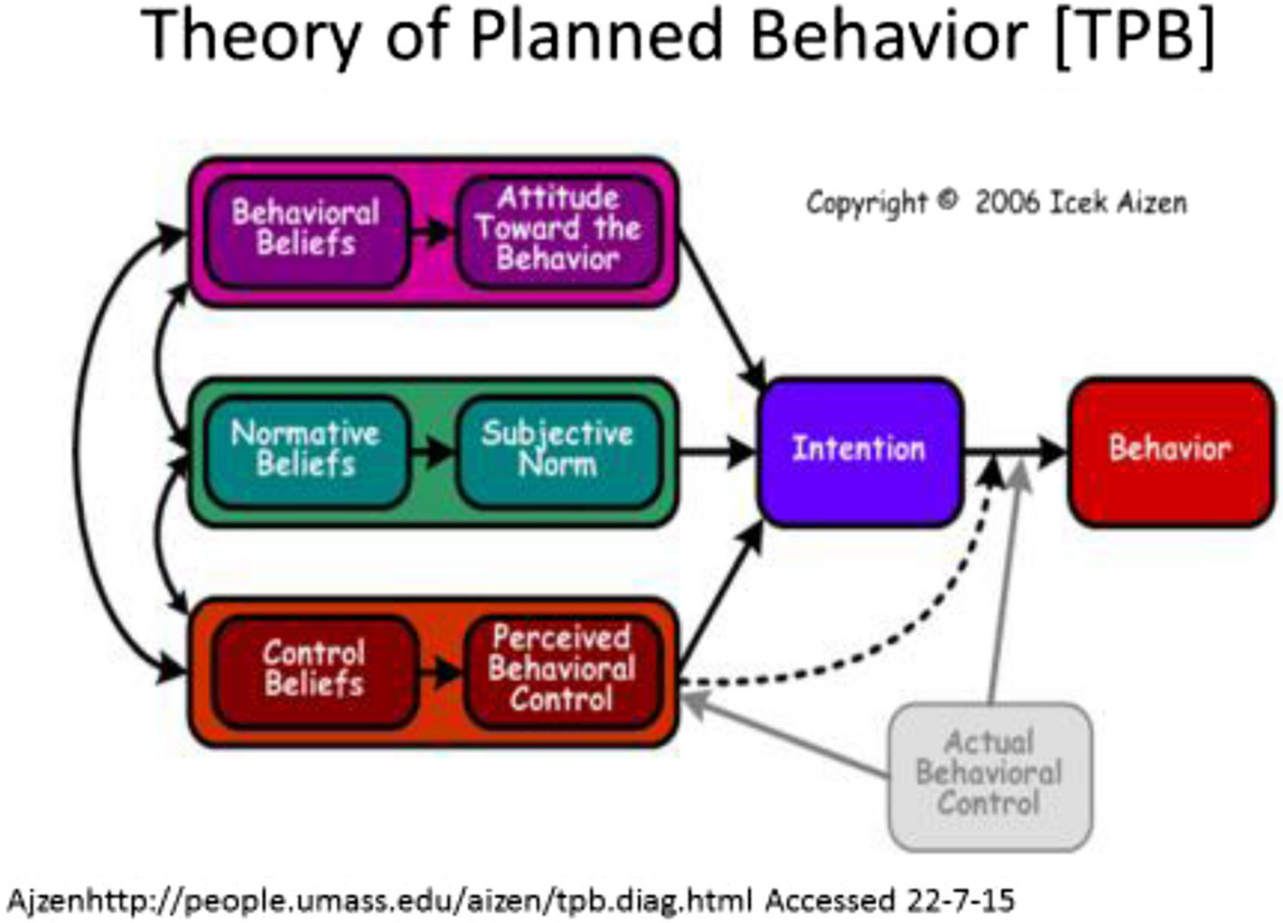
Theory of Planned Behaviour

This study was based on the Theory of Planned Behaviour (TPB) [19] to address the above deficit of theoretically based research in this area. The TPB explores the psychosocial influences on behaviour and provides an appropriate construct for identifying parents’ attitudes, normative influences toward and perceived behavioural control over the introduction of CFs. It allows identification of the determinants of behavioural intentions in situations where people do not have complete volitional control, or are not necessarily motivated to, or interested in, changing behaviour. The theory postulates that a person’s intention to perform a behaviour is the most important determinant of their action. Underlying the TPB are the antecedents of attitude, subjective norms and perceived behavioural control, corresponding salient beliefs which reflect an individual’s intention and subsequent behaviour [19]. See Fig. 1 [20]. In areas not previously studied the TPB recommends an initial qualitative elicitation study is undertaken to establish these cognitive foundations of the target population’s salient, most commonly occurring, beliefs [21]. Previous studies based on the TPB have successfully identified modifiable factors in the areas of parenting and child health, both in Australia and internationally [15, 16, 18].

The aims of this elicitation study were to: 1) identify first-time mothers’ salient beliefs with regards to the introduction of CFs, specifically behavioural, normative and control beliefs (TPB), 2) identify their sources of information about the introduction of CFs, and 3) explore first-time mothers’ knowledge of and attitudes towards National, NH&MRC, [2] and International, WHO, [1] recommendations. This paper reports findings from qualitative elicitation study, the first part of a larger study to identify the determinants of Australian parents’ intentions to introduce CF at six months. Findings from the larger study can be found in Hamilton and colleague’s papers [22, 23].

## Methods

### Study design

A qualitative study using a semi-structured interview guide was conducted through individual interviews and focus groups. The focus of the study was to identify factors influencing first-time mothers’ decision-making, to gain an understanding of mothers’ thinking, and generate new knowledge in relation to first-time mothers’ decision making regarding the timely introduction of CFs. Both methods, interviews and focus groups, involve participant explanation, provide insight into sources of complex behaviours and motivations and are appropriate in areas of limited research [24]. Interview dynamics place a burden on interviewees to explain themselves to the interviewer and are a source of private beliefs; in group discussions group members query each other and explain themselves to each other revealing perceptions of more public beliefs and knowledge [25]. Data collection method was dependent upon each participant’s availability.

## Ethical approval

The study was conducted within the ethical guidelines of the Declaration of Helsinki [26] with approval obtained from the Queensland University of Technology Human Research Ethics Committee (No. 0800000012). Voluntary participation was by informed written consent with assurances of confidentiality and anonymity. Authors bracted their own beliefs when developing the research and semi-structured interview questions. As these were theoretically based the theory was the focus of the questions rather than individual researcher’s beliefs or interests.

### Sample

For diversity of opinion on the research question a purposive, convenience, sample of 21 metropolitan first-time mothers self-selected to participate. Eligibility criteria included aged 18 years or older, able to read and converse in English and being a first-time mother to at least one child aged between 6 and 12-months.

### Procedure

Participants were recruited through advertising at local playgroups and childcare centres in Brisbane, Australia, and electronically through the University community email list. Upon a parent’s indication of interest in participating it was determined whether they would be able to join a focus group, or participate in an interview at their home. Some mothers choose to be interviewed when their child had an afternoon sleep. Those interested in participating in a focus group determined where and when the group would meet. Demographic data were collected from all participants. Study data were collected qualitatively using a semi-structured interview guide developed based on the TPB constructs [21] to direct the interviews to ensure similar data about salient beliefs were collected. To ensure data trustworthiness and that comprehensive valid data were collected, multiple discussions were undertaken and data collection continued until theoretical sufficiency was achieved and confirmed [24].

### Semi-structured interview questions

An interview guide was developed following the advice from Francis [21]. The aim of this was to ensure all participants were able to discuss the same topics and to ensure data collection followed the theoretical framework of the TPB. There were four focuses: behavioural beliefs, subjective norms, perceived behavioural control and knowledge of WHO and/or NH & MRC recommendations [1, 2].

1. Focus One - Behavioural Beliefs
  What do you believe were the advantages or benefits of introducing solids?
  What do you believe were the disadvantages or risks of introducing solids?
  Is there anything else you associate with introducing solids at a particular age?
2. Focus Two – Social referents and sources of information
  Were there any individuals or groups that approved or encouraged you to introduce solids?
  Were there any individuals or groups that disapproved or discouraged you from introducing solids?
  Where do you go for information about introducing solids?
  Has any particular information you have influenced how you manage fever?
  How accurate do you think the sources of information are/which sources do you think are accurate or reliable?
3. Focus Three – Perceived behavioural control beliefs
  What factors or circumstances made it easy for you to introduce solids?
  What factors or circumstances made it difficult for you to introduce solids?
  Are there any other issues that come to mind when you think about introducing solids?
  How old was your infant when they first received solids?
  How did you know when to introduce solids?
4. Focus Four – Knowledge
  What do you know about the current recommendations for the age at which solids are to be introduced?
  How do you feel about these recommendations?

### Data analysis

In accordance with the TPB, data were analysed thematically within the theoretical constructs, a method used in TPB [19]. Thematic analysis is a method which identifies, analyses and reports patterns or themes within the data [27]. Phases included familiarisation with the data; generation of initial codes; searching for themes or patterns; themes and patterns reviewed; and, themes and patterns defined and named. Assumptions consistent with the TPB were held with regards to the nature of the data. The second and first authors, both experienced qualitative researchers, independently identified the themes, categories and sub-categories for two transcripts. These were checked and deemed to be consistent. Remaining transcripts were analyzed by the second author according to the themes and categories identified.

## Results

A total of 21 mothers participated in the study, 13 in interviews and seven in the three focus groups. Focus groups consisted of *n* = 2; *n* = 3; *n* = 3 participants. Most (96.3 %) were in a partnered relationship. Many had a university degree (76.8 %) and a total weekly income greater than $5,000. Infants had commenced CFs from three and a half months (3.6 %) to six months (25.0 %) with most during the fifth month (42.9 %) and a quarter (28.6 %) commencing CFs during the fourth month of life.

Consistent with the theoretical framework of the TPB the themes, which are grounded in the direct experience of the participants, were deducted into three distinct constructs:

- Behaviour beliefs: beliefs about the advantages and disadvantages to the introduction to CFs at six months,
- Normative beliefs: beliefs about the expectations of important others and motivation to comply with these beliefs,
- Perceived control beliefs: barriers preventing and motivators encouraging mothers.

### Behavioural beliefs

A number of beliefs about the advantages and disadvantages to introduction of CFs at six months were identified by the participants, which reflected their attitude toward the behaviour.

#### General advantages to CFs

Mothers identified various advantages to the general introduction of CFs. Some were concerned about their baby’s growth and weight gain, especially if they were exclusively breastfeeding, and introduced solid food to supplement their breast milk. For example, one mother said:

*I don’t know if this is proven or not, that breastfed babies tend to be smaller sort of babies. So I think that that’s a benefit now that I’m onto solids she has sort of picked up a bit on weight (P3).*

Not only did the mother’s appreciate the added nutritional benefit of the CF, but also a reduction in the personal demands of breastfeeding. They perceived it as helpful and a relief to *‘share the load*’ of feeding their baby. Another mother explained an unexpected benefit from CF:

*She was fuller for longer so she wasn’t as attached to me…because she was feeding every three to four hours right up to when she was four and a half months. And then when we started her on that breakfast feed there was – I would get a couple of hours extra (P13).*

Some mothers liked the idea of their baby being the *‘first’*, or developmentally advanced and decided to introduce CF as early as possible, as *‘it is good to eat’*. For some it was purely seen as a fun and exciting activity which their baby and they could enjoy together. Interestingly, improved night sleeping patterns was another reason for CF introduction, however, in reality they found that it had minimal influence on night sleeping duration. A number of mothers did not identify the introduction of CF as advantageous, but rather a *‘natural progression’* in the baby’s development, and just *‘something that was going to happen.’*

#### General advantages to the timing of introducing CFs (at six months)

Most of the mothers interviewed had commenced solid foods prior to the recommendation of six months, yet for those who did wait some key advantages were identified. For one mother the decision to wait until six months was easy, and once the baby was introduced to CF, the process was straightforward. Another mother commented about her pleasant surprise with her baby’s interest and eagerness to eat: .

*we started her with rice cereal. And she loved it, the first week I couldn’t believe how much she was eating! (P20) I only tried about three or four different foods, fairly slowly. She actually loved it and loved feeding it off a spoon and swallows it straightaway (P15).*

One of the mothers had adopted the Baby-led Weaning approach [10], which endorses a developmental approach to the introduction of CFs, with babies commencing directly on soft finger foods, rather than pureed consistencies. This mother’s experience reflects positively on this approach:

*It was fun. I took a totally different approach to everyone else. Basically, he didn’t do purees, went straight to finger foods so it was very amusing watching. I found it fascinating. I was a little bit nervous because I did go to straight to finger food and not puree so there is obviously a bigger chance or higher risk of gagging and things like that which is awful to watch a child do. But, yeah, I found it enjoyable because of all the different tastes and the textures and everything (P5).*

Overall, for those mothers who waited until six months, they key advantage expressed was a simple transition to food, with the baby eagerly accepting the change without hesitation or difficulty. Those who shared this belief and waited until six months found the introduction of CF an easy, pleasant experience.

#### General disadvantages to introducing CFs at six months

Whilst many mothers did not identify any disadvantages in their experience of introducing CFs, there were a few themes which emerged suggesting that four months may have been too early. For example, one mother said:

*I started [name suppressed] at about four months old, on just some Farex but she couldn’t figure out how to swallow it she kept pushing it out with her tongue. So it sort of went on for a while and at first I thought it was just never going to happen But now we’ve hit six months she just wolfs it down now (P7).*

This mother’s experience would suggest that whilst the baby was showing an interest in eating, developmentally she was not yet ready, anatomically and physiologically, to actually eat. Other disadvantages identified by the participants were concerns regarding food allergies, mess and extra work. Parents had some awareness that early introduction of CF may trigger certain allergies however this was rationalised by considering family history and friends experiences. They avoided highly allergenic foods at the beginning, such as, nut based foods and dairy if they had concerns. One mother’s concerns:

*The only risks that I was concerned about was [name suppressed]’s allergies. Because just with the eczema we did notice that she has a tendency to flare up with any unusual material So we were a little bit concerned about that (P20).*

### Normative beliefs

The subjective norm within the TPB refers to how much an individual perceives important others’ expectations of them to perform the behaviour and their motivation to comply with these expectations [19]. There were two groups from whom mothers’ perceived social pressure: peer groups which included friends, family and health-care professionals including doctors and child health nurses.

#### Peer group influence

While some parents had clear intentions to wait until six months before introducing solid foods, others gave it very little serious thought or planning. For those mothers, decisions were easily influenced, particularly by the peer group and friends and family, as expressed by this mother’s reason for commencement:

*I hadn’t really thought much of it and then another friend of mine with a baby the same age, and she was introducing it, so I thought I would have a go (P19).*

A number of participants expressed a level of pressure from family members to introduce CF early, especially from the older women in their lives. For some parents this pressure caused them to commence early, whereas for other’s health professional or guidelines were perceived more important.

*And I had heard some of my other friends in the mums group saying they had started their babies as early as four months. So I kind of felt …behind I suppose …I was hearing all the great results that they had… But understood that my paediatrician knew best so I had to wait until she was at least six months of age before I could attempt (P20).*

*they (grandparents) were told to start giving babies orange juice at three weeks old…that it didn’t hurt you guys so you have nothing to lose they were really pushing the introduction of solids (P25).*

Importantly, the majority of participants stated they valued their family and friends beliefs regarding introduction to solids above that of health professionals or printed literature.

#### Health professionals and WHO recommendations

Some mothers with a good understanding of the WHO recommendations expressed feelings of guilt and ‘feeling like a bad mother’, even if they commenced early on health professional advice, as the mother comments below (her infant had significant gastro-oesophageal reflux).

*I guess I felt like a bad mum because I knew the World Health Organization recommended six months. Working in child care, that was always my idea – about six months. And I felt like I was doing something wrong and it might be potentially harmful to my son. ..(P7).*

There was minimal discussion regarding disadvantages of waiting until six months. The five month age was identified as challenging for one mother who had chosen to wait until six months to introduce CF:

*the last month and a bit came to be quite difficult because she was clearly interested in what was happening and what was around. It was that tension of “the World Health Organization says not to start until six months” …and that was conflicting to the cereal packets stuff like that at the shops … they all say from four months. And I had heard lots of other people say they were feeding their kids from four months. .. (P5).*

These statements reflect the complexity of this decision for mothers - multiple influences and information sources which contradict or support the recommendation to introduce CF at six months.

### Perceived control beliefs

The study explored factors motivating or acting as a barrier to the introduction of CF at six months.

#### Motivators relating to the timing of introducing CFs at six months

Participants identified several factors which had a significant influence over their choice of timing regarding introduction of solid foods. Cues demonstrated by their infant were predominant motivators, such as when the baby appeared very interested in family food, and keen to eat. In these cases, mothers felt they were depriving the infant by withholding the food until six months.

*I actually was trying to listen to her cues, I figured because she was taking an interest in us eating then it was worth trying. And if she had shown no interest, then even if people would have said …I probably would have resisted (P28).*

*I was adamant from the information I got that I would not introduce until six months. But she just wanted it, she had developed to the point that it was the next step for her (P11).*

The belief that ‘every baby is different’ and trusting maternal judgement were discussed by a number of mothers. For instance:

*And then even trusted experts and friends said “well yes that’s what they say but judge the child, look for these signs.” And in the end a lot of it had to be your own judgement I think (P19).*

### Barriers relating to the timing of the introduction of CFs

An interesting factor influencing decisions to introduce CFs was the notion of ‘eating off the shelf’; if the baby was already on formula this appeared to diminish the conviction of waiting until six months to introduce CFs. This was compounded by the marketing of commercial baby food products, which are labelled ‘from 4 months’ or ‘all ages’ adding to parental confusion.

*Because in my mind I’d weaned her from gaining nutrition from me weeks and weeks ago. She was getting supplements from me, with two breastfeeds a day, but she was a formula fed bub and that’s something I buy off the shelf in the supermarket…which is what I do for Farex and what I do for her other foods (P16).*

Some parents believed that the supermarkets would not be legally able to sell baby food products from four months if it was harmful to the baby’s well-being.

*Because I know that they are not going to sell it to you marked as seven to ten months if they can’t have it…I figure they are the experts and they’re not willing to be sued over an allergic reaction in a child. So they would make sure it’s the right stuff [food] that they can have at that age (P11).*

However, not all parents were convinced about the trustworthiness of the baby food companies:

*I have always wondered how they can get away with that [labelling baby food’from 4 months]. I would have thought there were laws against it. I didn’t understand it, and I don’t think it is right. They shouldn’t be able to say “from four months plus” because they are just like any other company that’s marketing their product. Just because they say from four months on doesn’t mean that’s the best thing for your child…I think it’s really deceptive and it shouldn’t be that way (P7).*

Barriers to introduce CF at six months were food labelling, perceived trustworthiness of food labelling of baby food products.

### Information sources

There is an abundance of information available to parents on how to parent. First-time parents are more likely to be influenced by others. Therefore sources of information to assist decision-making re the timing of introduction to CF were explored. Findings will enable specific and targeted education for mothers.

Many mothers voiced significant preference for information from peers who had recently been through the food introduction process, as they could understand and remember what it was like, in comparison to friends or family who had not so recent an experience.

*But I did have one good piece of advice… they said to me “don’t take any advice from someone who hasn’t had children in the last three years”. Because things seem to change, new research has come on board and that someone within the last three years would know a great deal more than someone who perhaps has had their children fifteen, twenty years ago (P20) .*

This quotation reflects the importance of the peer group and friends and family’s attitude and experiences regarding the introduction of CFs. Once parents had made the decision to introduce solid foods, they stated they explored various information sources to guide the how, what and where. Many mothers valued particular reference books, such as Robin Barker’s *Baby Love*, as useful guides regarding which foods to introduce, textures and quantities. Other parents referred to the internet for assistance, although a number of mothers stated that it was easy to become overwhelmed with information if you refer to multiple sources.

*I also did initially a little bit of internet research and the only really interesting thing that came out of that was that there is so much information presented in more and more confusing ways (P19).*

One mother aptly described her preference for ‘human’ sources of information, rather than literature, guidelines and the internet. She referred to it as ‘mothering by community’:

*But most of the time now I mother by community as opposed to by books and internet because I just think there is too much information out there, you know, and it can confuse you. You are constantly at risk of feeling like you are not doing the right thing as a mother (P5).*

Overall the mothers’ experiences reflected a complex and multi-faceted decision-making process with regards to the timeliness, types and approaches to the introduction of CFs.

## Discussion

The factors influencing mothers’ decision making were their salient beliefs regarding the advantages and disadvantage of introducing CFs at six months, the influence of important others (normative beliefs) and the perceived control which mothers’ had over their decision making. Mothers most valued information about the introduction of CFs from peers who had recently introduced CF. Mothers initially determined when, then sourced information about how, what and where. Overall, the study found that waiting until six months proved challenging for first-time mothers, despite knowledge of the WHO recommendations and initial desire to comply with these.

### Beliefs

A number of knowledge beliefs and attitudes expressed by participants influenced their decision making process regarding when they introduced CFs to their infant. The nutritional adequacy of breastfeeding exclusively between the ages of 4–6 months was questioned by a number of mothers, perceiving that breastfed babies were smaller and could do with the extra caloric support of CFs. It can be argued that society’s perception of a healthy baby is not consistent with a normal weight range, due to the high rates of artificially fed infants [12]. With childhood overweight and obesity increasing rapidly [8] and breastfeeding a demonstrated protective factor against this significant health concern [2], it is critically important that the impact of exclusive breastfeeding for the first six months is addressed and promoted. Further, when a parent correctly perceives a normal weight range for an infant or child, they can implement measures to bring the infant into a normal range [28]. Conversely, when infants are overfed to conform to societal pressure harmful consequences may emerge. Health care professionals can utilise appropriate growth development tools, such as the WHO Child Growth Standards [29], to reassure parents of the normal growth rate of breastfed infants, and challenge societal perceptions to promote healthy and normal nutrition and subsequent weight ranges.

An important maternal belief system highlighted by the parental discourse was the notion of ‘nutrition off the shelf’. Those mothers who were already feeding their infant manufactured foods (infant formula) perceived that the progression to solid foods early would not cause any more harm, as they were not breastfeeding. This is consistent with a similar study which indicated that mothers not breastfeeding exclusively until six months were more likely to introduce CFs prior to four months [30], earlier than their mixed-feeding or breastfeeding counterparts. Adding to this belief was the conflicting information on baby foods labelled ‘From 4 months’ or ‘4-6 months’. Some mothers believed that this labelling implied safety and suitability, despite it being against health professional and WHO recommendations. Yet, other mothers were not as convinced and more sceptical of the food manufacturers. This mixed finding has been substantiated in one other quantitative study which found that food labelling was a normative influence for some [23], yet this was not the case for all [31]. Knowing this belief system, and the relationship between artificial feeding and early introduction to CFs, provides health care professionals with clear insight to provide anticipatory guidance to families of the health risks of early introduction, specifically those already feeding ‘off the shelf’.

Mothers were all aware of the WHO recommendation, but few were aware of the comparative NH & MRC guidelines. Therefore, WHO recommendations were used throughout the study. However, this in and of itself, did not appear to strongly influence their decision making. Conversely, a 2010 study in the United Kingdom (UK) exploring factors influencing mothers’ decision making to wean their infant found those mothers experienced conflicting influences when deciding when to introduce CFs to their infant [31]. ‘Conflicting cues’ was identified as the overarching theme in decision making, and later introduction of CFs was associated with a focus on the 6 month recommendation as important and not the perceived infant cues [31].

Horodynski and colleagues [28] qualitatively explored low-income mothers’ decision-making regarding the timeliness of the introduction to CFs. This study examined knowledge and practice regarding the American Academy of Paediatrics recommendation, which was to introduce between 4–6 months, thus ‘early’ was prior to 4 months. Interestingly, they also found that though mothers were aware of the recommendations but they were not necessarily convinced that they applied to all infants [28]. Our well educated, middle-class cohort knew about the recommendations, yet did not necessarily believe they applied to their own infant or family unit as each mother-baby dyad was seen as unique. While our participants could reiterate the broad aspects of the recommendation, when it came to specific understanding regarding potential harms or disadvantages these were not well understood. Targeting this lack of knowledge regarding the harms of introducing earlier than recommended has been suggested as a more effective approach to education [28], rather than awareness of the benefits alone.

### Subjective Norms

The peer-to-peer groups of the mothers involved in the study were identified as having a strong influence over behavioural intention and subsequent behaviour. Peers who had recently or were currently parenting young babies were seen as a valuable source of information and knowledge. Mothers cited that seeing the ‘good results’ or perceiving their baby ‘as falling behind’ if they were not conforming to the group norm was a frequent theme. Group norm has been shown in the wider literature to influence women’s behavioural intention in other aspects of infant nutrition [23] and breastfeeding continuation [32]. Two mothers in the participant group cited a trusting, known relationship with their doctor as the active influence which persuaded them to adhere to the WHO recommendations, despite the peer group influence. Trust and a known health care provider have been cited broadly in the literature as having a positive influence on health behaviours and outcomes for women and families [33–35]. Thus, parental education and support is best provided within this context, in preference to fragmented episodes of care.

### Perceived behavioural control

Mothers’ control over the introduction of CFs prior to six months was controlled by baby cues and perception of infant hunger. Infant hunger or readiness cues were also frequently cited as a reason for commencement of CFs prior to six months. This is consistent with other studies exploring why mothers introduce solid foods early [28, 30]. Despite the mothers stating initially that they were ‘adamant’ about following the recommendation, they conceded their ‘baby just wanted it.’ Behaviours such as showing interest and reaching and grasping for family foods were cited cues for knowing baby was ready. Yet, when the actual commencement of CFs was explored in detail, mothers who introduced CFs at four months stated their infants required ‘encouragement’ and that a lot of the initial foods (such as baby rice cereal) were pushed back out of the mouth with the tongue. Yet, for the few mothers who introduced CFs close to six months the transition was positive, straightforward and eagerly accepted by the baby. Mothers in this study were not aware that developmentally babies around 4 months old are interested *in everything* parents are doing, yet that does not mean they are actually ready to do a particular task [10]. For example, they may watch a parent intently folding the washing, however this does not mean the baby developmentally is able to do this task yet. As many of the parents in this study identified their baby’s interest in food as the key trigger for commencement, health professionals may consider this lack of thorough knowledge about true signs of readiness as an area for targeted education, support and further research.

Overall, the parents’ dialogue revealed a superficial understanding and awareness of the recommendation to exclusively breastfeed for six months; however the actual and detailed significant health benefit of this recommendation was poorly understood as demonstrated by their comments and behaviours.

### Information sources

Mothers were aware that knowledge changed over time and sought advice from friends and relatives who had young children and had therefore been through the process of deciding when to introduce CFs in line with latest thinking. An earlier study [35] demonstrated that mother’s use many sources to gain information about feeding their infant. Initially many seek information from health professionals, however, this source of information reduces as time goes by to be replaced by information from relatives and family; this information source becomes more important and remaining consistently high during childhood [35]. Recent research demonstrates that expectant mothers highly value information provided by health care professionals more so after their baby is born than earlier [36]. Some mothers in this study also identified the trusting relationship with a health professional as important in guiding decision making, and this provides opportunity for health professionals to convey evidence-based information in a timely way. However, it is essential that all health professionals provide evidence-based information. In the 2010 UK study participants reported inconsistent advice regarding timeliness from health care professionals [31].

### Strengths and limitations

This study makes an important contribution to understanding the decision-making processes mothers use when deciding to introduce CF including knowledge of the WHO and NHMRC recommendation to introduce CFs at six months of age. The sample was sufficient to reach theoretical sufficiency. However, mothers were recruited from South-East Queensland, Australia. Findings, therefore, may not be generalizable to all settings. However they provide a deeper understanding of the factors which influence parents’ intentions and thus behaviours. The findings are qualitative, and are thus not intended to be generalised in the scientific sense, however theoretical generalisation is possible. To ensure appropriateness of the research method the RATS guideline for BioMed Central were adhered to [37].

## Conclusions

A number of modifiable factors which health professionals can address to improve the current low compliance with the WHO recommendation to exclusively breastfeed for six months have been identified. First-time mothers in this study did not demonstrate a good understanding of the rationale behind the recommendation, nor did they understand fully the signs of readiness of infants to commence solid foods. Factors that assisted waiting until six months were a trusting relationship with a health professional whose practice and advice was consistent with the recommendations; and, an infant developmentally ready for CFs and accepted them with ease and enthusiasm when delayed until six months. Barriers preventing parents complying with the recommendations included subjective and group norms, peer influences, infant cues indicating early readiness and food labelling inconsistencies.

This study enabled identification of specific motivators and barriers for health care professionals to target through innovative evidence-based parenting education and support to improve the health of young Australians. Specific theoretically based educational interventions (e.g., based on the TPB), designed to change behaviours should target one or more of these factors and evaluate subsequent changes in intention and behaviour. Findings may assist in reducing the global problem of overweight and obesity in our young children.

## Abbreviations

WHO: World Health Organisation
NH&MRC: National Health and Medical Research Council
CF: Complementary foods – defined in the background
TPB: Theory of planned behaviour
UK: United Kingdom
